# Tapping into Charge Storage with Operando-XPS Using a Multi-Layer Graphene Coplanar Capacitor and an Ionic Liquid Mixture

**DOI:** 10.1021/acs.langmuir.6c01458

**Published:** 2026-05-26

**Authors:** Ezgi Kutbay, Merve Taner Camci, Burak Ulgut, Oliver Hofft, M. Said Ergoktas, Coskun Kocabas, Sefik Suzer

**Affiliations:** † Department of Chemistry, 52948 Bilkent Univeristy, Ankara 06800, Turkey; ‡ 52996 Turkish Energy Nuclear and Mineral Research Agency, Ankara 06510, Turkey; § Institute of Electrochemistry, Clausthal University of Technology, Clausthal-Zellerfeld D-38678, Germany; ∥ Department of Physics, 5292 University of Bath, Bath, BA2 7AY, United Kingdom; ⊥ Materials Department, Manchester University, Manchester M13 9PL, United Kingdom

## Abstract

X-ray Photoelectron Spectroscopy under bias tracks surface population and electrical potentials on graphene electrodes with an ionic liquid solution, composed of one anion (TFSI^–^) and two cations (DEME^+^ and Rb^+^). As bias increases, ions are enriched at the analyzed vacuum/graphene interface due to electrosorption, and the measured binding energies shift due to both bias and ion screening. The relative population of Rb^+^ increases, but that of DEME^+^ decreases to maintain electroneutrality. After prolonged biasing, induced currents increase by more than 1 order of magnitude, attributed to a significant contribution from electrosorbed, smaller, and mobile Rb^+^ cations, causing a sizable asymmetry with respect to the polarity of the bias. The evolving relative concentrations of Rb^+^ and DEME^+^ allow us to elaborate on the dynamics of the ionic structure inside the electrolyte. These findings bring a new understanding of the surface composition of electrified interfaces in terms of anion and cation balance.

## Graphical abstract

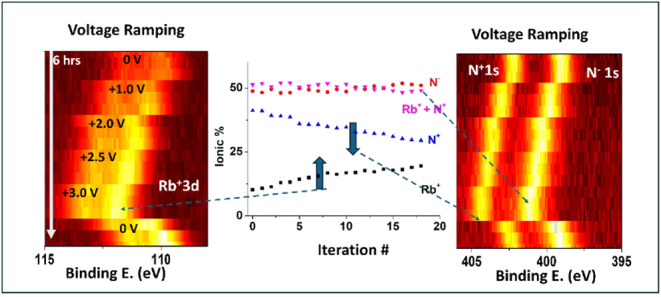

## Introduction

Control of ionic motion is vital for energy storage and harvesting systems, such as batteries, fuel cells, and supercapacitors (electrical double-layer capacitors).^1–6^ Ionic liquids (ILs), having large operating electrochemical windows and negligible vapor pressure, have emerged as promising alternatives to conventional aqueous and organic electrolytes.^7–11^ For electrode materials, different forms of carbon, like graphite, graphene, and carbon black, have been studied, since suitable porous structures can be tailored to optimize charge storage and retrieval from the electrolyte.^11–14^ Together, these attributes make IL–carbon interfaces a compelling platform for high-performance electrochemical devices. However, ILs consist of large anions and cations and hence exhibit slow dynamics, which poses an important drawback to be mitigated. Despite their advantages, relatively large anions and cations lead to slow ion dynamics and limit rate performance. This drawback has motivated strategies aimed at enhancing ionic mobility, including the use of ionic liquid mixtures and the introduction of smaller ionic species.^15–19^ It was shown that mixing two ILs that share a common cation but differ in anion size improves device performance by up to an order of magnitude, an effect attributed to modified ion packing and reduced transport barriers near the electrode interface.^20^

Supercapacitors are increasingly deployed in energy management applications, including regenerative braking, load leveling, and high-rate energy recovery.^21–23^ To improve energy density, asymmetric electrode configuration designs have emerged, in which one electrode undergoes faradaic (battery-like) reactions while the counter electrode remains a conventional carbon-based capacitor.^24^ Although such hybrid configurations offer higher specific energy, their reliance on redox processes introduces performance limitations—such as reduced rate capability and long-term cycling stability—which diminish their suitability for fast-response or long-lifetime applications.^9^ These limitations motivate alternative approaches that enhance capacitance without faradaic chemistry. Achieving a molecular-level understanding of electrochemical interfaces requires experimental techniques capable of resolving both chemical composition and local electrical environments under operating conditions.

Recent efforts to better understand, at the atomic/molecular level, of the various chemical and physical components affecting the dynamics of electrochemical systems include advanced electrochemical, spectroscopic, and microscopic techniques, accompanied by theoretical and simulation methods.^25–35^ To this end, advanced electrochemical measurements have been combined with spectroscopic and microscopic methods, often supported by theory and simulation. Recent examples include studies integrating electrochemical analysis with NMR and Raman spectroscopy to elucidate anion-size effects on Li-ion transference in polymer electrolytes.^36^ X-ray photoelectron spectroscopy (XPS) is a unique technique that has been utilized by us and others for the simultaneous probing chemical states and local electrical potentials within working electrochemical devices.^37–41^ Moreover, XPS is chemically selective and quantitative, and Raman is neither.

On the other hand, XPS has previously revealed substantial deviations from bulk electroneutrality at graphene/ionic liquid interfaces under applied electric fields. Wang et al. reported significant anion/cation ratio deviations of up to 20% near the interface.^42^ Earlier, slightly smaller values were also reported by us for a similar system.^43,44^ Herein, we extend our investigation on two multilayered graphene (MLG) electrodes with IL electrolyte, containing a dissolved inorganic salt for tracking competitive electrosorption and time-dependent reconfiguration in this mixed-cation ionic liquid electrolyte. The canonical IL, DEME-TFSI, we have been using^39,43,46,47^ is also well-suited for capturing compositional variations, since it has one nitrogen atom per formula of its anions and cations, which are also well-separated in the binding energy scale. Ion ratio variations can easily be implemented by dissolving readily available cationic-TFSI salts within this IL, where a certain fraction of the large quaternary nitrogen cations is replaced by small alkali cations while preserving the anions’ identity. In the present contribution, we dissolved ∼10 atomic % Rb-TFSI salt with this IL and used it as the electrolyte of a two-electrode electrochemical device. Similar to the neat ionic liquid, this mixture has very low vapor pressure and is UHV compatible.

By exploiting the composition of the distinct interfaces formed on the two electrodes, we obtain complementary insights into ion-specific electrosorption, local potential developments, and their evolution under prolonged biasing. Beyond the application of biased XPS, the present work reveals how competitive electrosorption between a bulky ionic-liquid cation and a smaller, more mobile inorganic one leads to time-dependent and asymmetric interfacial reorganization.

## Experimental Section

The MLG electrodes are deposited on a porous polymer membrane, which acts both as a separator and a support for introducing the IL electrolyte. This setup forms a coplanar capacitor, where one electrode is biased and the other is grounded, enabling simultaneous analysis of electrode surfaces and electrolyte. To monitor the changes in interfacial composition, rubidium was chosen instead of lithium for XPS measurements because the Rb 3d photoemission signal is significantly stronger than that of the Li 1s level with AlKα X-rays. The Na 1s level has a large binding (and smaller kinetic) energy, making it difficult to faithfully quantify.^45^ Similarly, the K 2p level overlaps with the C 1s levels, of especially those belonging to −CF_3_ groups of the TFSI^–^ anion, hence it is also not so easy to quantify.

Our coplanar MLG device operates as a two electric double-layer (EDL) capacitor, where charge storage arises from the rearrangement of ions at the electrode–electrolyte interface without major chemical transformations, thereby reflecting only the intrinsic behavior of nonfaradaic capacitive interfaces, which is schematically shown in Figure 1. Experimental details are given in the Supporting Information (SI) ection.

**Figure 1:**
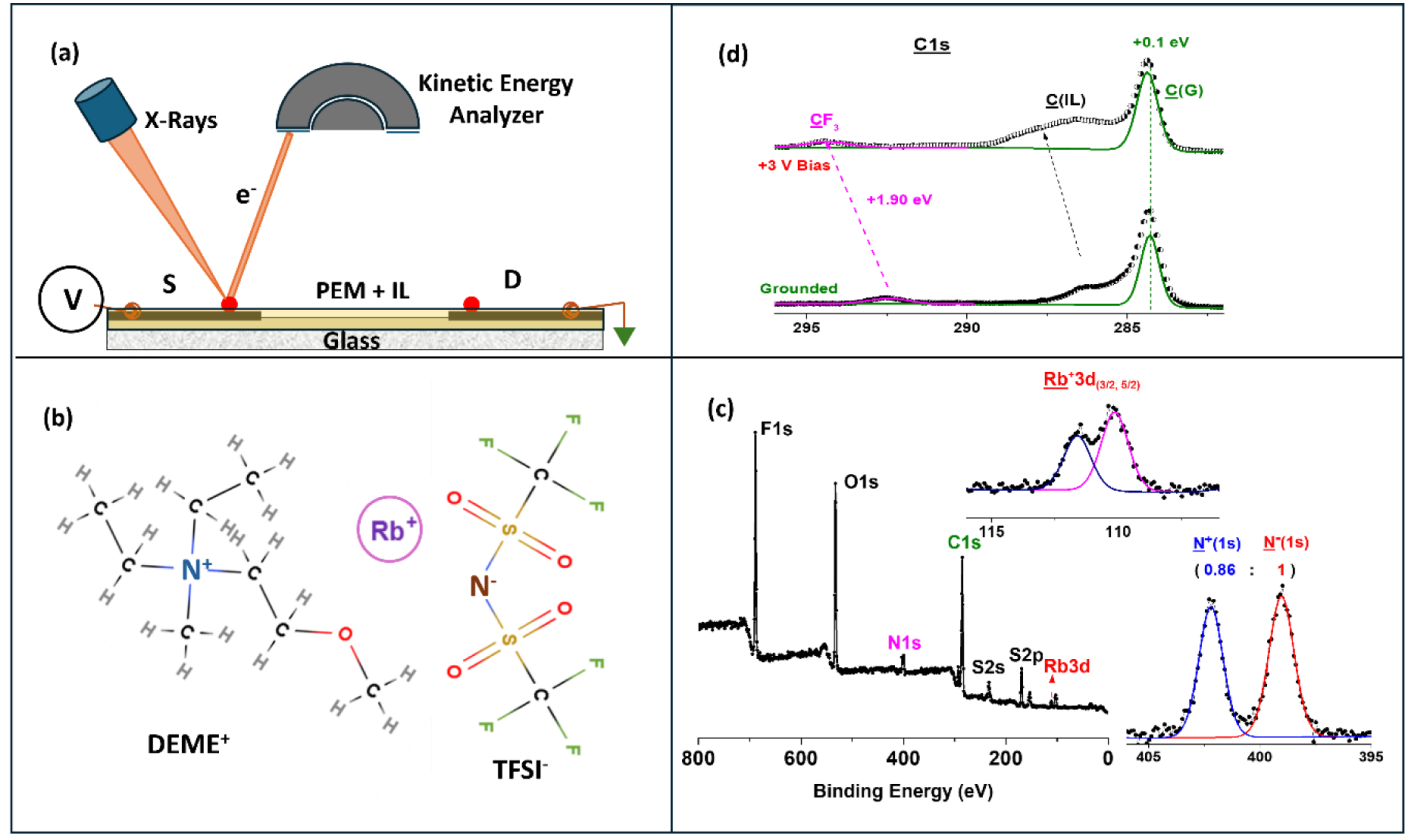
(a) Eye-level schematic of the coplanar MLG capacitor device with a source and a drain electrodes. (b) Chemical structures of DEME^+^, TFSI^–^, and Rb^+^. (c) XP survey and Rb 3d and N 1s regions spectra at the unbiased state, where, due to the presence of ∼10% Rb^+^, the observed N^+^/N^–^ ratio is smaller than 1 (∼0.86). (d) The C 1s spectra of the composite surface of the grounded electrode before and after +3.0 V biasing, emphasizing the fact that, whereas the single peak corresponding to the graphene carbon is almost unaffected, the peak due to −CF_3_ carbon undergoes a sizable shift, which is, however, less than the full +3 V bias.

Because the ionic liquid creeps and covers all accessible surfaces, it is already present on electrode surfaces before biasing (Figure 1c); hence, the C 1s region contains both graphene- and electrolyte-derived features. Under applied bias, the C 1s peak and other core levels associated with the electrode and electrolyte shift by different amounts, as shown in Figure 1d. The molecular structures of the electrolyte components are illustrated in Figure 1b. With a calculated volume of ∼14 Å^3^ (based on a 1.5 Å ionic radius), Rb^+^ is by far smaller than the bulky DEME^+^ cation (∼220 Å^3^) and the TFSI anion (∼200 Å^3^), which is expected to approach electrode surfaces more effectively. Therefore, one of the aims of this work is to evaluate how the presence of Rb^+^, a significantly smaller cation compared to DEME^+^, influences the interfacial charge distribution and electrosorption dynamics. Reproducibility of the data obtained has been corroborated by repeating the measurements on two additional similar devices.

## Results and Discussion

Within XPS, kinetic energies (K.E.) of the ejected photoelectrons are measured with high precision, and using Einstein’s formula^45^

(hν=B.E.+K.E.)

binding energies (B.E.) of atomic levels of the ejected electrons are computed, which is also schematically shown in Figure S4 in the ESI section. The X-ray photon’s energy is 1486.60 eV in our instrument. As shown in Figure 1c, the resulting spectrum consists of well-separated regions corresponding to the atomic core level peaks of the chemical moieties residing on the analyzed surface. The C 1s peak of the IL (−CF_3_) has a binding energy of 292.80 eV when the device is grounded from both sides, which translates to (1486.60 – 292.80 = 1193.80 eV) the kinetic energy of the ejected photoelectron. If we applied a +3 V bias, the kinetic energy of the photoelectron emitted by C 1s should decrease to 1190.80 eV (red shift), or exactly 3 eV, since the bias increases the measured binding energy. However, this does not happen, because the measured shift is not +3 eV but only +1.90 eV (Figure 1d). This reveals the fact that the IL medium reports less than the applied potential due to screening by the nearby anions, so that the electrical potential of the IL at that state and position is measured as +1.90 V; hence, +1.10 V has been shielded by the counterions. As such, recording the core level shifts under bias turns XPS into a local chemical voltmeter.^43,44,46^

Voltage-dependent data were recorded in scan mode at points near the electrified electrode (NE) and the grounded electrode (NG), as indicated in Figure 1a. The DC voltage was ramped stepwise from 0 V to +3 V and then returned to 0 V (shorted) to capture compositional changes, as well as charging, discharging, and relaxation processes, which are displayed in Figure 2.

**Figure 2:**
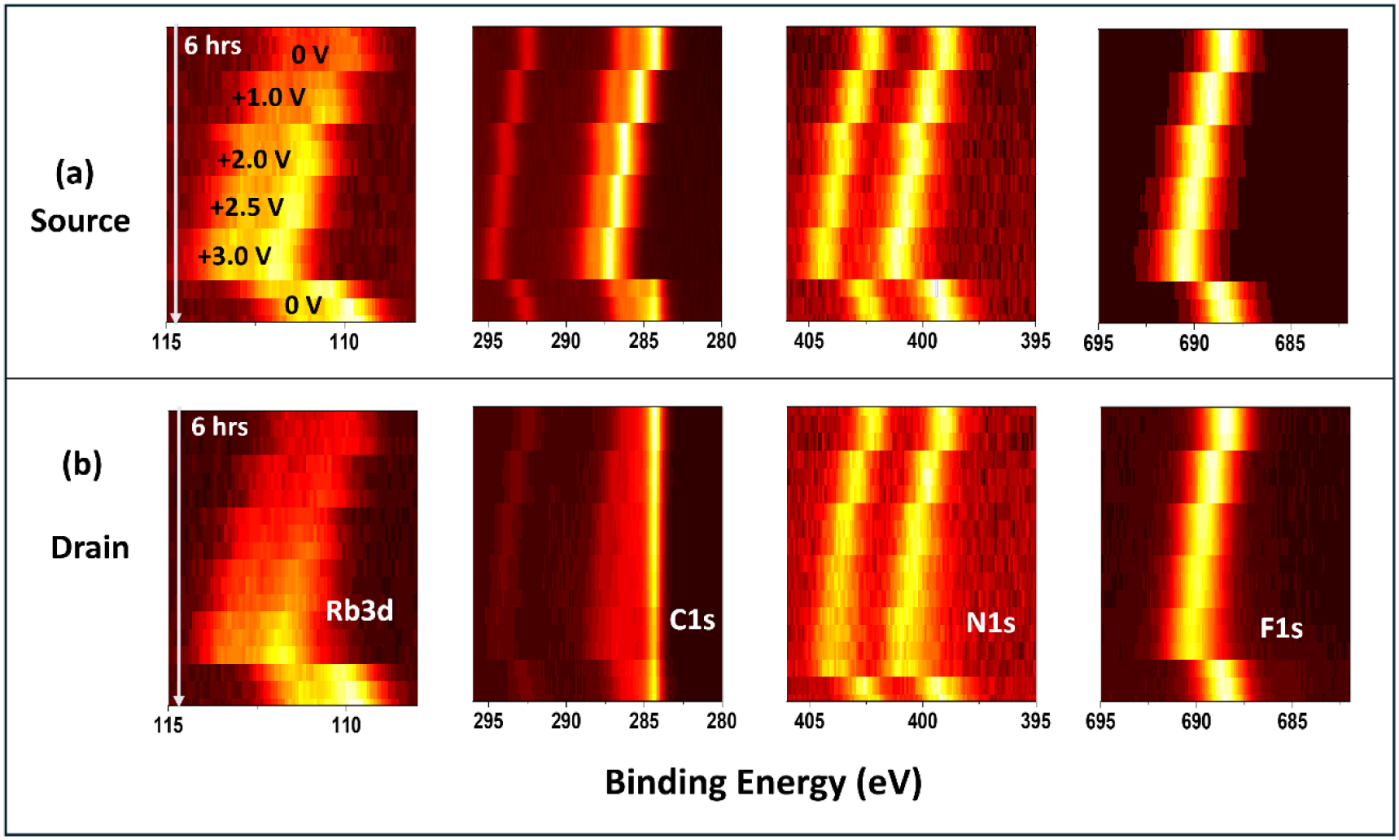
Heat-scale 3-D graphs of XP spectra of the Rb 3d, C 1s, N 1s, and F 1s regions were recorded sequentially for 6 h each, while the bias was ramped gradually by 1 V steps, up to +3 V, and shorted afterward. On (a) Source and (b) Drain multilayered graphene electrodes, respectively. The total duration of data gathering was 6 h each, corresponding to 18 min for each iteration.

At both the source and the drain electrodes, the Rb 3d, C 1s, N 1s, O 1s, and F 1s peaks of the IL solution gradually shifted to higher binding energy positions with increasing positive voltage, consistent with changes in their local potentials (Figures 2a and 2b). At the same time, their intensity increased at the expense of the graphene C 1s peak. Note also that at the grounded electrode, unlike the others, the position of the same C 1s peak of the graphene undergoes very little or almost no shift, since the electrode is grounded. Upon returning to 0 V, the binding energies slowly approach their baseline values, and for some species, the values briefly dipped below the baseline, which is consistent with the transient polarity reversal behavior when the system is shorted. This important finding was recently reported by us and is attributed to annihilating the fast electronic component of the positive bias upon shorting, while the slow negative ionic component of the electrical potential continues its decay.^46^ Measurements under negative potential ramping have also been performed to ensure that mirroring behavior was also observable. The recorded full XPS data of Figure 2, as well as those under negative voltage ramping, are provided in Figure S4 in the Supporting Information (SI) Section.

For quantitative analysis, peak areas are obtained by integrating the signal under the fitted peaks (rather than the raw data points), which revealed that the relative amount of Rb^+^ at both electrode surfaces increased with applied bias, while the signal from the DEME^+^-derived N 1s feature decreased. These trends are shown in Figure 3b and 3d on the two electrode surfaces, and the results of the detailed analysis are given in Table SI in the Supporting Information (SI). The total duration of data gathering was 6 h each corresponding to 18 min for each iteration.

**Figure 3:**
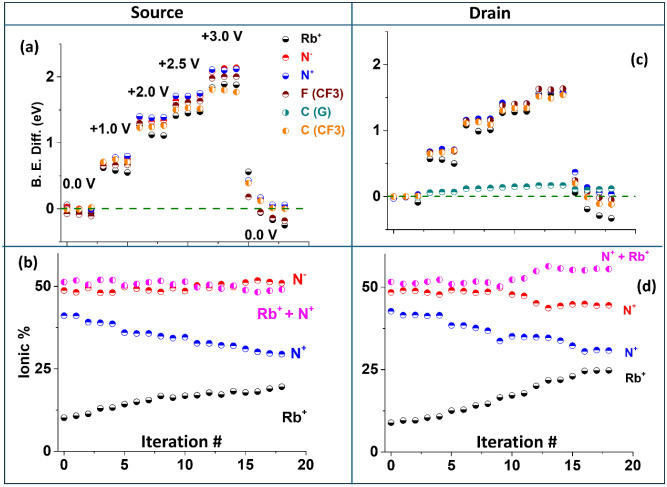
Extracted binding energy shifts from Figure 2 with respect to the grounded state (0 eV) (a) recorded on the source MLD electrode under gradually increasing positive bias. (b) Evolution of atomic percentages for Rb 3d, cationic N 1s, N^+^, and anionic N^–^ during the positive voltage ramping at the Source electrode. (c) Binding energy shifts with respect to the grounded state (0 eV) on the drain MLD electrode. (d) Evolution of atomic percentages for Rb 3d, cationic N 1s, N^+^, and anionic N^–^ during the positive ramping at point NG.

These large changes are attributed to the redistribution of cations and anions at the interfacial regions under applied potential. Therefore, probing the interfacial liquid layer adjacent to the electrode renders highlighting the dynamic restructuring of the EDL and enhanced ionic accumulation at the interface, rather than any permanent modification of the graphene electrodes, all of which are now uniquely evidenced via XPS measurements. Despite the observation of large compositional changes, the overall trends remain clear and unchanged, so that the relative TFSI^–^ signals (N 1s, O 1s, and F 1s) remained essentially constant, indicating that exchange between the two cations is the primary mechanism accommodating the changing surface charge. More importantly, the combined Rb^+^ and DEME^+^ (N^+^) content closely tracked the TFSI^–^ (N^–^) signal during the voltage ramp, indicating that charge neutrality is maintained at the interface within the limited depth of ∼8 nm probed by XPS.

The ability of the small, highly mobile Rb^+^ to complement the bulkier DEME^+^ supports a model where the double layer reorganizes throughout the entire graphene-bulk via ion rearrangement at the multitude of distributed IL-graphene interfaces, rather than through insertion between the layers. These reversible and polarity-sensitive shifts in core-level binding energies, with corresponding intensity changes, serve as direct spectroscopic evidence of potential-induced ion redistribution at the interface. By introducing a second, small inorganic cation Rb^+^ into the IL, we uncover a bias-polarity-dependent and history-dependent interfacial response that is absent in single-cation systems and cannot be inferred from equilibrium measurements alone. The dynamic nature of the relative intensities of Rb^+^ and N^+^ from DEME^+^ clearly shows that the ionic structure under bias is dynamic, even after the local potential appears to have settled. The two ions appear to move toward homogenization without making significant changes to the local potential.

After voltage ramping, the current response of the device, shown in Figure 4, increases by nearly 2 orders of magnitude, as also shown in the same figure. A similar device having only the neat DEME-TFSI ionic liquid, i.e., without Rb^+^, gave only 1 order of magnitude increase in current, as shown in Figure S2 in the SI section. As was also reported in our previous paper,^46^ we attribute this pseudocapacitive increase,^47^ among a multitude of processes, largely to electrosorption as a result of prolonged biasing, which can also render an increase in the electroactive area in the graphene electrodes.^33^

**Figure 4:**
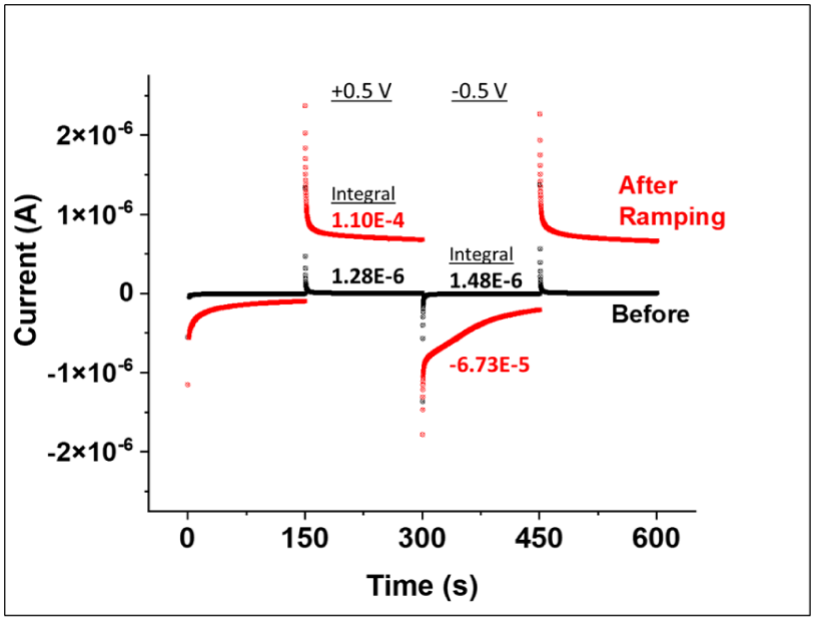
Current–time measurements taken before and after the voltage ramping process, showing the 2 orders of magnitude increase in the current after ramping. The integrated areas are also indicated.

Moreover, after 6 h of voltage ramping, an apparent asymmetry with respect to the polarity of the bias also sets in. This is evidenced by the observation that after imposing the first-0.5 V bias cycle for a duration of 150 s, during which cations are predominantly electrosorbed into the MLG electrodes, a much larger positive current passes in the following 150 s and +0.5 V cycles. The polarity-dependent asymmetry in current, coupled with the reversible shifts in surface ion composition, highlights the dominant role of the relatively more mobile Rb^+^ ions, causing also a relatively higher transference in modulating interfacial charge transport.

Importantly, both cationic species participate in the interfacial response: their sum approaches that of the N 1s signal from the anionic species, consistent with local charge compensation at the interface. All these observations reinforce the idea that enhanced current and the response probed by XPS stem mostly from electrosorption rather than structural change. XPS-derived insights point to a surface-level, field-driven electrosorption mechanism rather than bulk intercalation or irreversible structural modification of the electrode. Together with the bias- and history-dependent redistribution between the two cations, these results indicate competitive electrosorption accompanied by time-dependent, partially irreversible interfacial reconfiguration under bias.

Note also that we have not considered the obvious mixture, where the cations are the same but the anions are different, due to the fact that, in the past, we had carried out angle-resolved XPS investigations on mixtures of the two ionic liquids, DEME^+^-TFSI^‑^ and DEME^+^-BF4^‑^, where we found strong surface enrichment, layering, and/or island formation, which were not suitable for quantification of the compositional variations of anions and cations.^48^

## Conclusions

In summary, the close correspondence between the XPS-derived surface ion distribution and the observed electrochemical behavior supports a model in which surface-level charge balancing, rather than bulk ion intercalation, dominates. These results demonstrate how competitive cation electrosorption and time-dependent interfacial reorganization can be directly tracked operando in mixed-cation ionic liquid electrolytes using biased XPS, revealing strong asymmetries and irreversible effects not accessible through electrochemical measurements alone. Incorporation of a small alkali cation like Rb^+^ reveals new degrees of freedom in modulating charge storage behavior, opening a path toward rational electrolyte design for asymmetric or directionally selective electrochemical interfaces. The size disparity enables Rb^+^ to more effectively penetrate the interfacial region, thereby exerting a stronger influence on the local electrochemical environment and modulating the charge distribution and electrosorption behavior at the electrode–electrolyte interfaces.

## Supplementary Material



## Data Availability

The data supporting this article have been included as part of the Supporting Information section, with experimental details, data, and figures.

## Notes

Details of materials and methods used, emphasizing especially on the electrical biasing of the devices used in this work, together with additional data on electrical current, local electrical potential variations, and chemical composition (PDF)
