# Supplementary material for: Tapping into Charge Storage with Operando-XPS Using a Multi-Layer Graphene Coplanar Capacitor and an Ionic Liquid Mixture
Source: Langmuir. 2026 May 26;42(22):15765–70. doi: 10.1021/acs.langmuir.6c01458 (PMC13262036; doi:10.1021/acs.langmuir.6c01458)
Supplement: Supplementary file 1 [file la6c01458_si_001.pdf]

Supporting Information

Tapping into Charge Storage with Operando-XPS using a Multi-Layer-Graphene Coplanar Capacitor and an Ionic Liquid Mixture

Ezgi Kutbay,[a] Merve Taner Camci,[b] Burak Ulgut, [a] Oliver Hofft,[c] M. Said Ergoktas,[d] Coskun Kocabas[e] and Sefik Suzer[*, a]

[a]Department of Chemistry, Bilkent University, 1. Cadde, 06800 Ankara, Turkey

[b]Turkish Energy Nuclear and Mineral Research Agency, Ankara 06510, Turkey

[c]Institute of Electrochemistry, Clausthal University of Technology, D-38678 Clausthal-Zellerfeld, Germany.

[d] Department of Physics, University of Bath, Bath, BA2 7AY, United Kingdom

[e]Materials Department, Manchester University, Manchester M13 9PL, United Kingdom

[*]Corresponding author (suzer@fen.bilkent.edu.tr)

Experimental Details

As electrodes multilayer graphene samples are grown on nickel foils (Alfa Aesar) using chemical vapor deposition at temperatures ranging from 850 to 1000 °C at ambient pressure. A mixture of H_2, Ar and CH_4 gases is used during the growth with flow rates set as 100 sccm, 100 sccm, and 30 sccm respectively, and the growth time is 5 mins. This procedure enables fabricating self-standing multilayer graphene films having 300 to 600 layers. Two 5x5 mm MLG film electrodes, separated by 5 mm, are transferred onto the Porous Polyethylene Membrane (PEM), which is placed on a glass slide and the ionic liquid is introduced below the membrane, which eventually permeates and covers the electrodes. The electrolyte solution consisted of ~90% (by weight) Ionic Liquid [*N*,*N*-Diethyl-*N*-methyl-*N*-(2-methoxyethyl) ammonium bis (trifluoromethanesulfonyl) imide] DEME-TFSI and ~10 % Rb-TFSI salt. Both chemicals were purchased from Io-Li-Tec and used for fabricating the co-planar devices. A schematic representation of the device, together with an SEM image of the MLG are given in Figure S1.

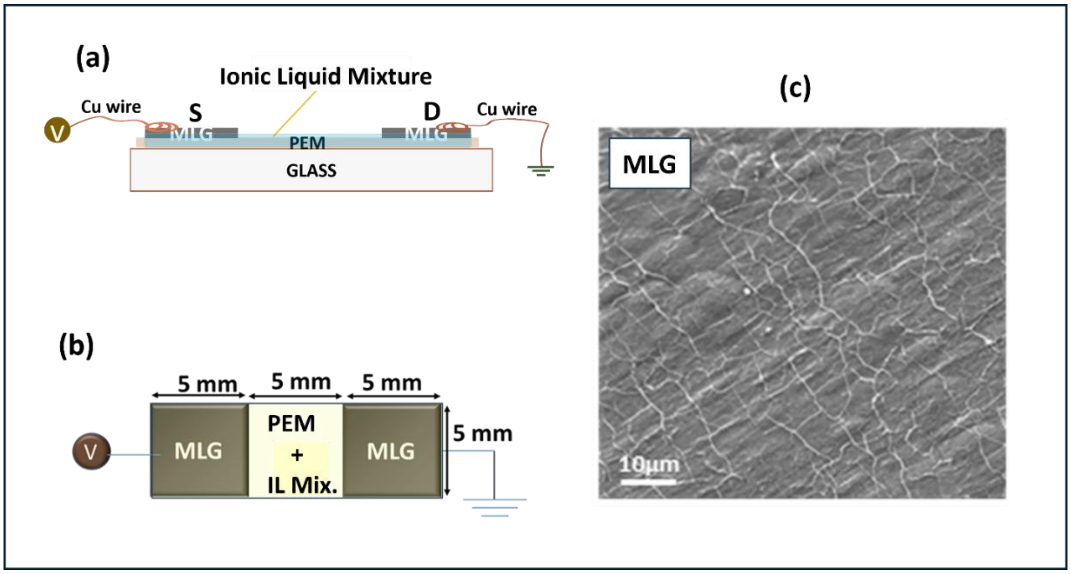

Figure S1. The Co-Planar Capacitor Device having an Ionic Liquid Mixture as the Electrolyte; **(a)** Side and **(b)** Top Views. **(c)** Secondary Electron Microscope Image of the pristine MLG surface.

A Thermo-Fisher K-Alpha X-ray Photoelectron Spectrometer with a monochromatized photon energy of 1486.6 eV has been used to collect data for all measurements. External bias is applied to one of the MLG electrodes using a Keithley 2400 Source-meter, and the other electrode is grounded. After introducing the device into the UHV system of the spectrometer, the system including the device is heated to 80 °C overnight to remove residual water and other volatile impurities. XP Spectra, both with and without external bias, as well as the induced current, have been recorded simultaneously. XPS data processing is carried out using the Avantage software package provided by the manufacturer.

Measurements

a- Current Measurements

As also mentioned within the main text, biasing also caused a noticeable increase in the apparent capacitance (C = Q/V) of the device, which is computed from the integrated current (accumulated charge = Q) after dividing by the voltage step (-0.5 to +0.5 V =) of 1 V, as shown in Figure 4 in the main text, the data of which is also reproduced in Figure S2 (a) below.

A similar change was also reported in our previous publication, where the device of similar dimensions and MLG graphene electrodes had only the neat DEME-TFSI, as the electrolyte.[1] After

14 hours of cycling under +2 and -2 V biasing with one-hour intervals, one order of magnitude increase in the capacitance of the device was recorded. The data from that paper is reproduced in the same Figure S2(b) for meaningful comparison.

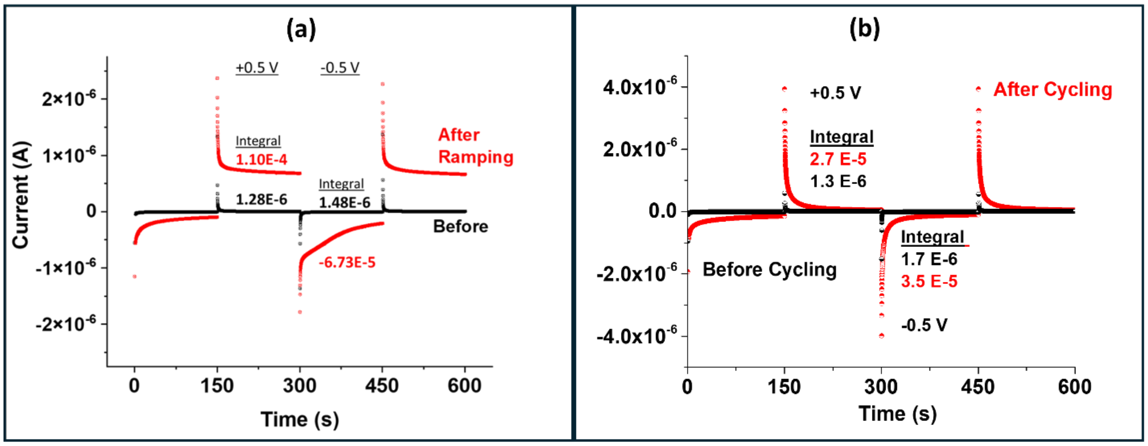

Figure S2. Current measurements under -0.5 V and +0.5 V bias for a duration of 150 s each. **(a)** The present device, which has the ionic liquid mixture (~10 atomic % Rb- and DEME-TFSI), where the initial currents are shown with olive data points, which increase after application of the 6 hours Biased Ramping for a duration of 30 minutes (1800 s) and becomes asymmetric (orange). Application of +2V bias for the same duration also causes an increase in currents and creates asymmetry in the opposite direction (purple). **(b)** The current measurements for a similar device, having only the neat DEM-TFSI as the electrolyte, before and after ± 2V cycling for 14 hours, which is adopted from Reference 1.

As is evident from both figures, the current passing through the system increases about one order of magnitude during the ramping/cycling processes for the device having only the neat DEME-TFSI. However, for the device having the mixture of 10% Rb+ cations, nearly two orders of magnitude increase in the current was recorded. We attribute these current increases, among a multitude of processes, largely to electrosorption as a result of the prolonged biasing, which can also induce increase in the electroactive area in the graphene electrodes. Moreover, having a small cation clearly increases it even more.

b- XPS Measurements

A typical XP Survey Spectrum recorded on top of the MLG electrodes with the IL electrolyte, containing ~10% (by weight) Rb-TFSI salt dissolved in it is shown in Figure S3, together with all peaks of the IL solution are visible. XPS is a strong chemical analysis tool, hence identity of all constituent atoms of the IL, except for hydrogen, are represented as corresponding peaks at certain

binding energies, well separated in the energy scale.[1,2] Moreover, chemical signatures of the atoms are also resolved by the corresponding shifts (chemical shift) in that scale. Accordingly, whereas only one fluorine peak bonded to carbon (-CF_3), one oxygen peak bonded to sulfur (SO_2) and one sulfur peak is observable, two separated nitrogen peaks are observable corresponding to the anionic and cationic moieties. In the C1s region, there are several overlapping peaks representative of the solid graphene electrode with those of as the electrolyte cations, together with another well separated peak where the carbon is bonded to the strongly electronegative F atom (-CF_3) of the TFSI anions. In the N1s region there are two well-separated peaks with unequal intensities; (i) representative of the TFSI anions, designated as N^- and (ii) another one belonging to the DEME cations, designated as N^+ are present at the lower and the higher binding energies, respectively, and their area ratio is 0.86:1.00. The other smaller cation is represented by the Rb3d spin-orbit doublet peak, the chemical composition of which adds up to that of the cationic N1s peak to satisfy the electroneutrality of the electrolyte medium.

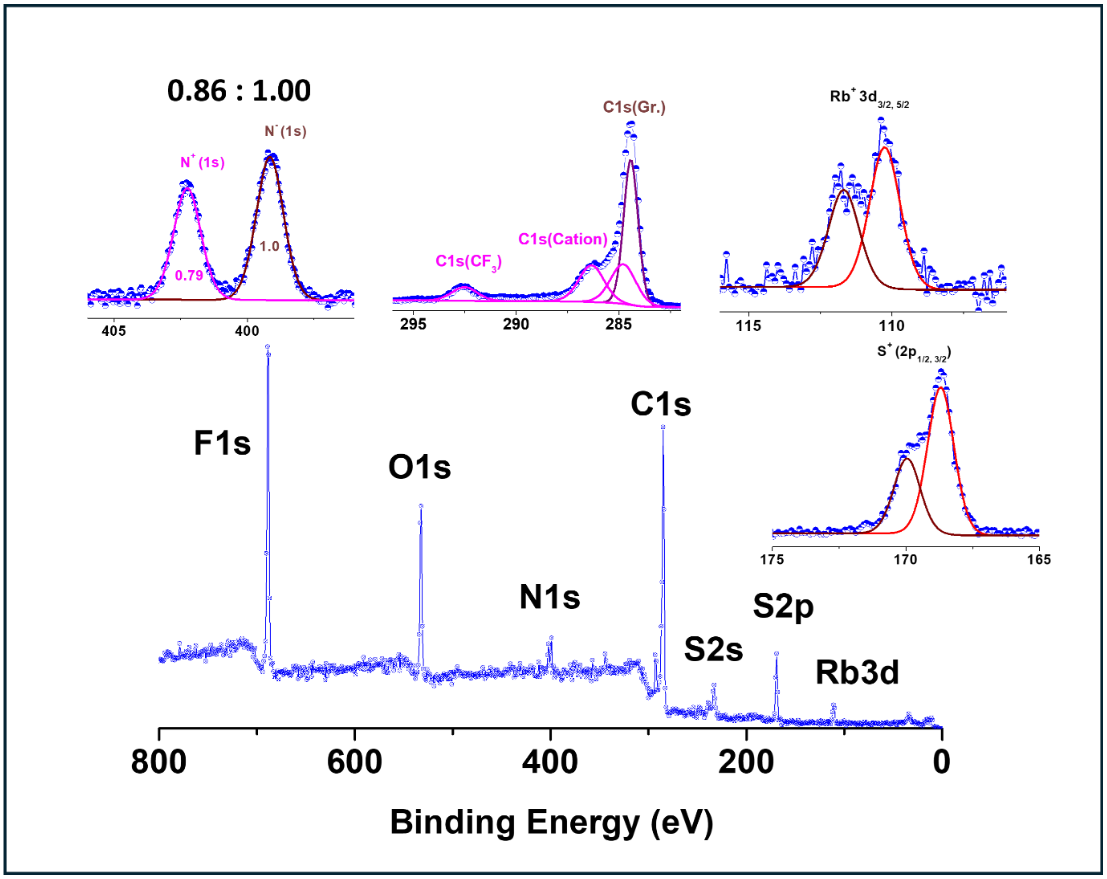

Figure S3. Survey XP Spectrum and the individual atomic regions, recorded on one of the composite MLD Electrodes, consisting of solid graphene and liquid electrolyte.

Table S1. Chemical Composition of the MLD after Electrosorption

	Binding Energy (eV)	FWHM (eV)	Area (cps)	Atomic %	
$Rb3d_{5/2}$	110.4	1.45	700	0.5	0.5 Rb^+ Cations
C1s (Graphene)	285.0	0.80	13200	30	5.0 -C_6- Units
C1s (CF_3-IL)	292.9	1.15	2300	4.6	2.3 TFSI Anions
N1s (Anion)	399.5	1.36	2120	2.5	2.5 TFSI Anions
N1s (Cation)	402.6	1.30	1800	2.1	2.1 DEME Cations
F1s (IL)	688.9	1.73	32200	18	3.0 TFSI Anions

This data enables us to estimate the molecular proximity of the graphene layers to the electrosorpted anions and cations with estimated average radii of 0.75 and 0.72 nm, and 0.22 and 0.20 nm^3 molecular volumes, respectively.[3] Although the density of the MLG is not known, we can assign a rough value of 2.0 g/cm^3, which is in between those of graphite (~1.7 g/cm^3) and HOPG (~2.3 g/cm^3). By taking the basic molecular unit of graphene as six carbons (-C_6-) and computing its molecular mass (6 x 12.011=) 72.066 g/mol, pure MLG is expected to have a molecular density of [2.0 g/mol / (72.066 g/-C_6-)] = ~ 27.8 $x10^{-3}$ moles/cm^3. This number can be translated to ~ 2.8 -C_6- molecular units /nm^3. A similar estimation gives ~1.4 $TFSI^-$ and molecular anions and $DEME^+$ (together with Rb^+) cations/nm^3, a very surprising result of almost one -C_6- unit per each type of ions. Moreover, assuming that the molecular volumes of the ions do not change upon sorption, we get 1.4 x (0.220 nm^3 + 0.200 nm^3) = 0.59 nm^3 as the total molecular volume of the ions, pointing out to a very tight packing. However, although we have no accurate experimental method of assessing the packing structure, we advocate that the high electrosorption must also be accommodated by a substantial volume increase.

The technique is also surface sensitive, since the created electrons carry electrical charge, and they are severely scattered by the condensed medium (solid and/or liquid) of the sample, hence only the electrons created within the top 0-8 nm survive with their unaltered kinetic energies.

Furthermore, to prevent additional scattering by the gaseous medium, all measurements are performed under ultra-high vacuum conditions.

c- XPS Measurements Under Bias

In addition to the powerful compositional analysis ability, XPS also measures the kinetic energy (K.E.) of the ejected photoelectrons with high precision. Using the well-known Einstein's photoelectric effect formula (**hν = B.E. + K.E**.), the technique gives the binding energy (B.E.) of the atomic level of the ejected electron as schematically shown in Figure S2. In the formula, **hν** is the X-Ray photon's energy, which is the monochromatized **AlKα** at 1486.6 eV, in our instrument.

Under standard analyses conditions, the sample is connected to the spectrometer's ground, as a result the Femi level of the solid/liquid sample is equilibrated with that of the spectrometer. When the binding energies are calculated they are also corrected with respect to the work function of the spectrometer, as also shown in Figure S4 (a). For the Multi-Layered-Graphene used in this study, the C1s level's binding energy is equal to 284.6 eV, when the device is grounded from both sides, which translates to (1486.6 – 284.6 =) 1202.0 eV kinetic energy of the ejected photoelectron.

If we apply a DC Bias of +2 V to the sample, the C1s photoelectron's energy is reduced to 1200.0 eV, since positive potential impedes the electron's kinetic energy (red shift), as a result, the measured B.E. is now increased by exactly +2.00 eV as shown in Figure S3 (b). A negative (-2 V) bias induces a corresponding -2.00 eV blue shift, resulting in a total energy difference of 4.00 eV between the two biasing conditions. The opposing shifts under ±2 V bias conditions produce a total energy difference of 4.00 eV between the two states, directly correspond to the applied external voltage and serve as a critical reference to validate the XPS measurement system under biased conditions. These shifts are referred to as trivial shifts and are used only to ensure the validity of the method.[2] However, similar measurements on the F1s peak representing the IL undergoes only 1.80 eV, i.e. less than the full 4.00 eV bias shifts, as shown in Figure S3 (c). This finding reflects the presence of an effective screening of (4.00 -1.80=) 1.20 V, created by the ions of the IL medium**.** Note that this is the most direct and relatively non-invasive method of measuring the local electrical voltage developments, **via XPS-The Chemical Voltmeter, not available by any other technique.** Unlike conventional electrical techniques, XPS can non-invasively reveal

internal voltage distributions and ion-induced screening effects at the molecular level. This makes it an exceptionally powerful tool for studying electrochemical systems, interfaces, and ionic environments.

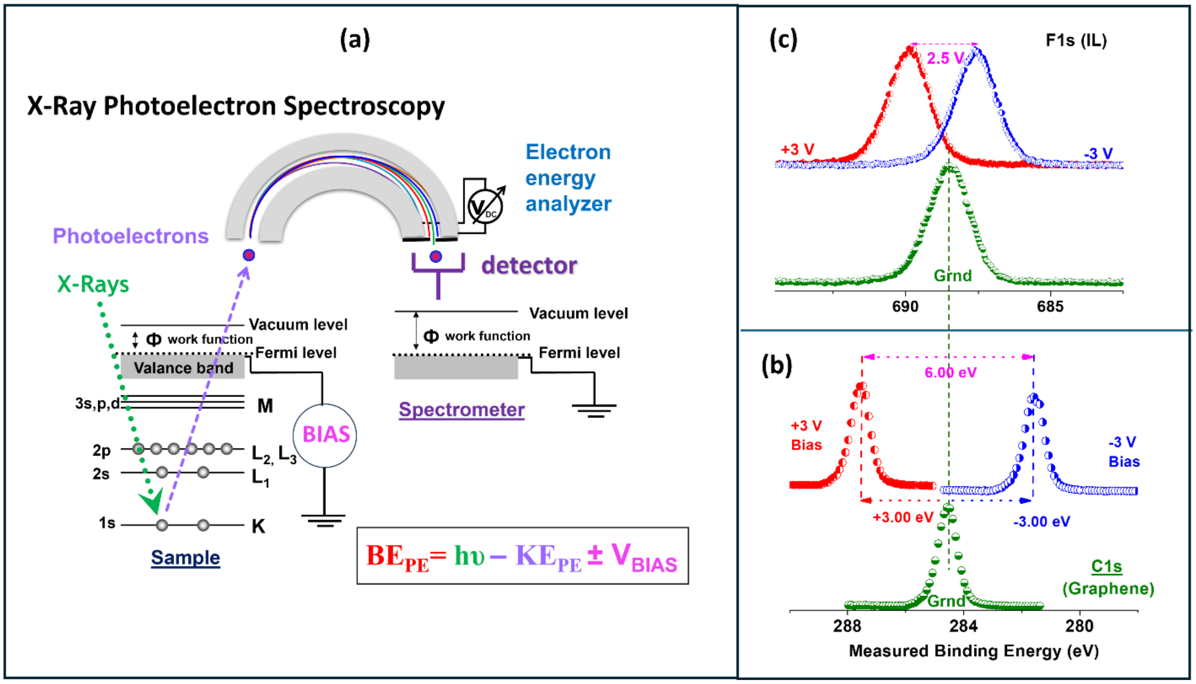

Figure S4 (a). Schematics of the XPS measurements. **(b)** C1s region of the pristine Multi-Layered Graphene Source Electrode and **(c)** F1s region of the Ionic Liquid after Iterations, recorded; when grounded (**green**) and under **+3 V** and **-3 V** bias, respectively.

The combination of positive and negative ramp data strongly supports the interpretation that XPS can resolve both the magnitude and dynamics of local potential changes with chemical and temporal specificity. Such chemically specific local voltage developments can also be measured/captured in a time-dependent fashion underscoring the temporal resolution of the chemical voltmeter approach, which has been outlined in the main text in Figures 2 and 3, and are redisplayed in Figure S4 for the data recorded on the Source MLG Electrode for 6 hours under increasing positive bias and similar data recorded on the Drain Electrode are presented in Figure S5, further validating the chemical voltmeter's ability to resolve subtle and dynamic electrostatic phenomena at electrode interfaces.

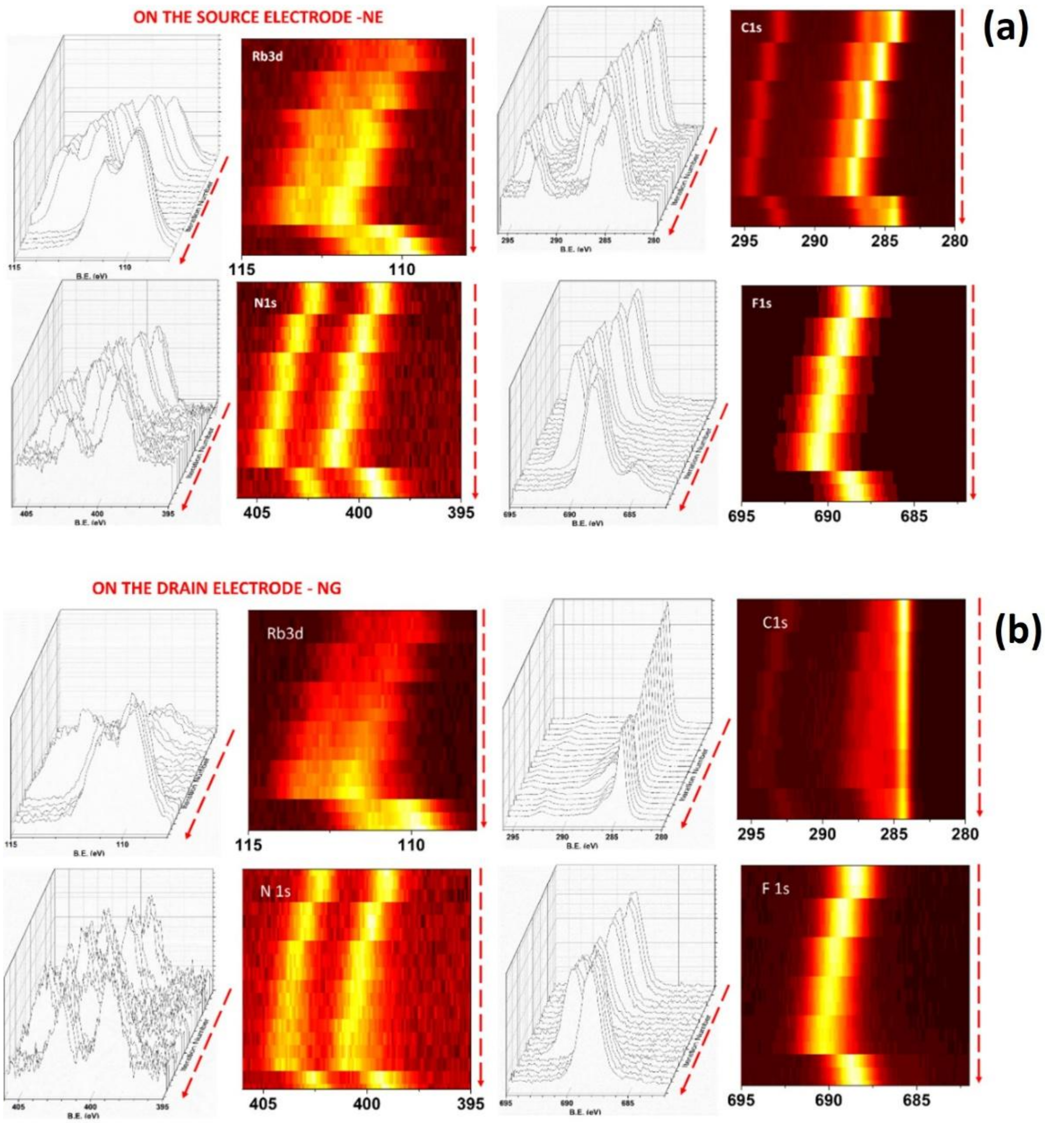

Figure S5. Two-dimensional (2D) time-resolved color map showing the evolution of Rb 3d, C1s, N1s and F1s core-level spectra on the (a) Source and (b) Drain MLG Electrode.

d- SEM Measurements Under Bias

Achieving and validating uniform voltage variations, along the surface of the coplanar capacitor device under bias, has been one of the important experimental tasks to overcome all along. Thanks to the spreading and/or creeping property of the ionic liquids, this is easily accomplished, with the help of the porous polyethylene membrane in between and underneath the electrodes. To validate this issue, we had mapped the voltage variations on the entire surface of the coplanar-capacitor, using AC modulated SEM measurements, as was discussed in detail in our earlier paper.[5]

If the energy of primary beam is low enough (~2 keV) the intensity of the emitted **Secondary Electrons' Yield can be modulated** by application of a small (3 V) AC- bias, which can be captured in a time resolved fashion, very similar to the present XPS measurements. This is related to the fact that low-energy secondary electrons are accelerated towards the detector at negative bias, but significant fractions of SE cannot escape the sample under the positive one. Although there is no chemical specificity as in the case of XPS, through its fast imaging property SEM data can be recorded within seconds to yield lateral images, as shown in Figure S5 below.

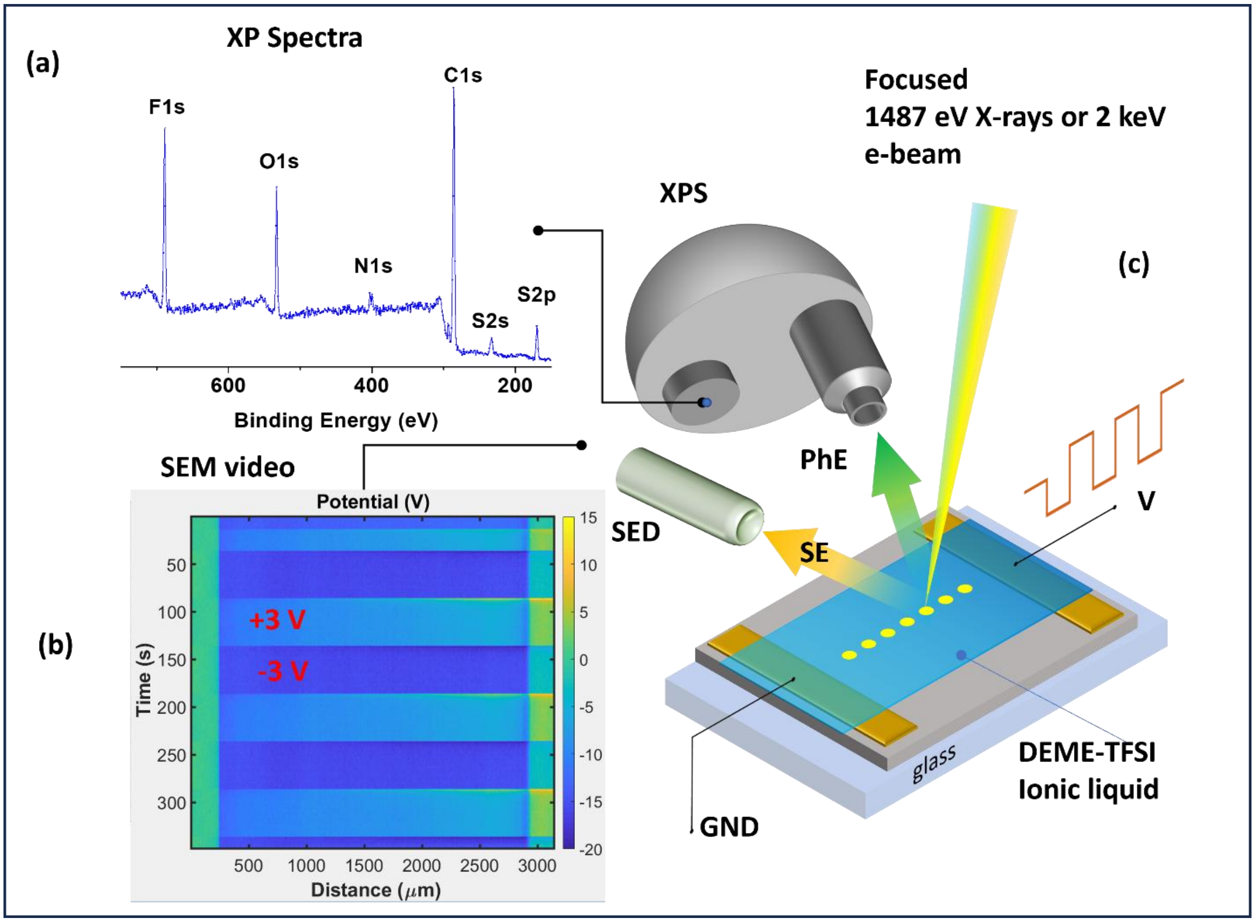

Figure S6. **(a)** XP Spectra of the neat DEME-TFSI Ionic Liquid. **(b)** Secondary Electron Yield Intensity-Time-Distance Map, obtained by averaging SEM video data along the spatial direction running parallel to the electrodes. The polarity of the AC signal is reversed every 50 seconds. **(c)** Schematics of SEM and XPS experimental set up of the same device but at two different labs. **SE** refers to Secondary Electrons, **SED** to the SE-Detector, the **PhE** to Photoelectrons.

To emphasize the uniformity of the observed polarization behavior, the SEM video data has been analyzed in the following way. Firstly, the time-dependent dataset of the SE intensity, SEY (x, y,

t) has been averaged along the dimension running parallel to the electrodes to yield a visualizable 2D map.

References

1 Kutbay, E.; Ulgut, B.; Kocabas, C.; Suzer, S., Unveiling Ionic/Electronic Contributions to the Potential Development of Electrical Double Layer Using XPS, *J. Phys. Chem. Lett.* **2025,** *16*, 8788-8784.

2 Briggs, D.; Seah, M. P. *Practical Surface Analysis, Auger and X-Ray Photoelectron Spectroscopy*, *Vol. 1, 2nd Eds.*; Wiley, Chichester, **1996.**

3 Taner Camci, M.; Aydogan Gokturk, P.; Başaran, M.; Ulgut, B.; Kocabas, A.; Kocabas, C.; Suzer, S. Dynamics of Potential Screening upon Electrification of Solid-Ionic Liquid Interfaces Probed by XPS. In *Encyclopedia of Solid-Liquid Interfaces*; Elsevier, **2024**; pp 661–680. https://doi.org/10.1016/B978-0-323-85669-0.00097-0.

4 Kim, Y.-J.; Matsuzawa, Y.; Ozaki, S.; Park, K. C.; Kim, C.; Endo, M.; Yoshida, H.; Masuda, G.; Sato, T.; Dresselhaus, M. S. High Energy-Density Capacitor Based on Ammonium Salt Type Ionic Liquids and Their Mixing Effect by Propylene Carbonate. *J Electrochem Soc* **2005**, *152* (4), A710. https://doi.org/10.1149/1.1869232.

5 Suzer, S.; Strelcov, E.; Kolmakov, A. Conparative Operando XPS and SEM Spatiotemporal Mapping of Ionic Liquid Polarization in a Coplanar Electrochemical Device. *Analytical Chemistry* **2021**, *93*, 13268-13272.
